# Coffee Cascara as a Source of Natural Antimicrobials: Chemical Characterization and Activity Against ESKAPE Pathogens

**DOI:** 10.3390/molecules31030403

**Published:** 2026-01-24

**Authors:** Merike Vaher, Olga Bragina

**Affiliations:** Department of Chemistry and Biotechnology, Tallinn University of Technology, Akadeemia tee 15, 12618 Tallinn, Estonia; merike.vaher@taltech.ee

**Keywords:** coffee cascara, phytochemicals, antibacterial activity, ESKAPE pathogens, polyphenols

## Abstract

Coffee cascara is a byproduct of coffee production traditionally used for infusions and animal feed. In this study, aqueous extracts of cascara from three different sources (Cas1–Cas3) were analyzed for their polyphenol and flavonoid content, as well as the concentrations of key individual bioactive compounds including caffeine, trigonelline, chlorogenic acid, and protocatechuic acid. Among the tested samples, Cas1 exhibited the highest total polyphenol (802.2 mg GAE/L) and flavonoid (134.7 mg QE/L) contents. The antibacterial activity of these extracts and an artificial mixture of the four compounds were evaluated against ESKAPE pathogens. Cas1 exhibited the most promising antibacterial effect, with minimal bactericidal concentration (MBC) values as low as 0.03 mg/mL for *S. aureus* and *A. baumannii*, and 0.26 mg/mL for *P. aeruginosa*. The artificial mixture, despite containing higher concentrations of the major compounds, exhibited reduced efficacy (MBC of 0.04 mg/mL for *S. aureus* and 0.15 mg/mL for *A. baumannii*, respectively), highlighting the superior activity of the native extracts. These results indicate that cascara extracts possess strong antibacterial activity, which correlates with their content of bioactive compounds, mainly polyphenols and alkaloids. The pronounced efficacy of the native extracts compared to the artificial mixture suggests that minor constituents in cascara may synergistically contribute to antibacterial effects. The present study highlights the potential of cascara aqueous extracts as natural multi-component antimicrobial agents, particularly against clinically relevant pathogens such as *A. baumannii*.

## 1. Introduction

The global expansion of multidrug-resistant (MDR) bacterial pathogens constitutes a major and rapidly escalating public-health crisis, undermining the efficacy of existing antibacterial therapies and complicating clinical management of infectious diseases. MDR is defined by the capacity of microorganisms to survive exposure to multiple antimicrobial agents, which leads to higher rates of treatment failure, prolonged hospitalization, increased mortality, and substantially elevated healthcare costs. MDR bacteria employ diverse resistance strategies, including enzyme-mediated antibiotic inactivation, target modification, decreased intracellular drug accumulation via efflux systems and porin alterations, as well as biofilm-associated tolerance and persistence phenotypes, all of which diminish the effectiveness of both first-line and last-resort agents. These mechanisms are particularly well exemplified by the ESKAPE group (*Enterococcus faecium* (*E. faecium*), *Staphylococcus aureus* (*S. aureus*), *Klebsiella pneumoniae* (*K. pneumoniae*), *Acinetobacter baumannii* (*A. baumannii*), *Pseudomonas aeruginosa* (*P. aeruginosa*) and *Enterobacter* spp. (*E. cloacae*)), which remain leading causes of nosocomial and difficult-to-treat community-acquired infections worldwide [1,2].

Compounding the clinical threat, the antibacterial discovery and development pipeline has failed to keep pace with the rapid emergence and dissemination of resistance. International assessments of the clinical pipeline highlight a persistent shortage of novel, innovative agents, especially those active against multidrug-resistant Gram-negative pathogens, and repeatedly call for prioritized research and coordinated investment to address critical gaps. In parallel, factors such as inappropriate or excessive antimicrobial use, limited diagnostic capacity for rapid pathogen and resistance identification, and inconsistent infection-prevention practices accelerate selection and spread of resistant strains across healthcare and community settings [3].

In this context, natural products, notably plant-derived secondary metabolites, represent a promising frontier for antibacterial drug discovery. Plants biosynthesize a wide array of structurally diverse compounds (e.g., phenolic acids, flavonoids, alkaloids, tannins, terpenoids) which can exhibit antibacterial activity through multiple mechanisms, including diverse modes of action: efflux pump inhibition, disrupting membranes, modulating quorum sensing, biofilm inhibition, and potentiating existing antibiotics [4]. Systematic reviews and recent natural-product surveys reveal numerous plant-derived compounds with in vitro activity against MDR organisms, including members of the ESKAPE group, and emphasize that substantial proportion of global plant biodiversity remains insufficiently explored for antibacterial potential [5,6].

Cascara, a byproduct of coffee production, consists of dried husk and pulp of coffee cherries and is traditionally used to prepare exotic drinks. The coffee pulp alone corresponds to approximately 28% of the coffee fruit on a dry weight basis, while the outer skin—approximately 12% [7].

The use of cascara has long been a traditional practice in coffee producing regions, and its use has recently begun to gain popularity in the Western world. According to the EU Commission Regulation, dried coffee cherry pulp is allowed to be used on the EU food market [8]. The cascara contains about 50% of carbohydrates, 10% protein, 20% fibers, 2.5% fat, and 1.3% caffeine, and small amounts of phenolic compounds. The major classes of phenolic constituents identified in cascara were flavanols (catechin and proanthocyanidins), hydroxycinnamic acid derivatives, flavanols and anthocyanidines [9] and chlorogenic acid as the predominant phenolic compound [10]. It was shown that coffee pulp is a potential valuable source of antioxidants and phenolic compounds which should not be discarded as waste [11].

A recent study presented in a published article reported that cascara contains two novel flavonoid glycosides (dihydromyricetin glycoside and dihydromyricetin rhamnosylglycoside) in addition to three alkaloids (caffeine, trigonelline, theobromine) and phenolic acids (gallic, protocatechuic, and chlorogenic acids) [12]. Furthermore, pectic polysaccharides were successfully extracted and characterized from selected coffee cascara pulp samples [12]. Consequently, cascara is the potential natural source of various bioactive compounds. In vitro studies have confirmed several potentially beneficial effects attributed to coffee cascara as a whole product, such as antioxidant, anti-inflammatory, antibacterial, adipogenic and lipolytic effects [11,13,14,15].

Building on the diverse bioactive composition of cascara, including polyphenols, flavonoids, alkaloids, and pectic polysaccharides, this study evaluated the antibacterial activity of aqueous extracts from three cascara sources against clinically relevant Gram-positive and Gram-negative pathogens. Additionally, the content of the key bioactive compounds, such as caffeine, trigonelline, chlorogenic acid and protocatechuic acid, were also quantified. While previous studies have reported chemical profiling and general antimicrobial activity of cascara, direct comparisons between native cascara extracts and defined mixtures of their major constituents at equivalent concentrations remain limited. Here, we subsequently compare activity of the extracts with artificial mixtures, formulated at equivalent concentrations and provide insights into the contribution of minor constituents and synergistic effects in enhancing overall antibacterial efficacy.

## 2. Results and Discussion

### 2.1. Total Soluble Solids of Cascara Extracts

To present the results of the bactericidal concentration based on soluble solids, the total soluble solid content of all cascara extracts was determined (Table 1). The results obtained were similar, ranging from 23.94 to 28.37 mg/mL.

### 2.2. Total Polyphenol and Flavonoid Content

Polyphenols are a diverse group of secondary plant metabolites synthesized to defend against environmental stressors, pathogens, and UV radiation, making them vital to the plant’s survival [16]. In coffee production, the coffee cherry pulp (cascara) is particularly rich in these compounds, including phenolic acids and flavonoids, as they are highly concentrated in the coffee cherry outer layers [17].

Total phenolic and flavonoid contents of cascara from three different producers are presented in Table 2. Both compounds were highest in the Cas1 extract, accordingly 802.2 mg gallic acid equivalents per liter (GAE/L) and 134.7 mg quercetin equivalents per liter (QE/L), being about two times higher than in the Cas2 extract, which demonstrated 403.5 mg GAE/L and 57.3 mg QE/L, respectively. These results are consistent with the findings by Rahmavati et al. They found that the total phenolic content of the cascara brew from different regions varies from 4.53 to 24.64 mg GAE/g, whereas the total flavonoid content varies from 0.83 to 4.07 mg QE/g [18]. Among the flavonoids, cascara contains a particular high amount of catechins (catechins, epicatechins) and condensed tannins or proanthocyanidins formed from these compounds [19,20].

Since the antioxidant activity is closely associated with the total phenolic content of the extract, it can be concluded that the antioxidant activity decreased in the following order: Cas1 > Cas3 > Cas2, likely due to the different coffee varieties, climatic conditions, and coffee processing method used.

In the wet (washed) process, the coffee cherry pulp is separated from the beans prior to drying; the cascara is collected fresh and dried as a separate material. In the dry (natural) process, the coffee cherries are dried directly in the sun without removing the husk. This is a long-term sun-drying process which leads to the degradation of phenolic compounds or, in the case of fermentation, is partially transferred to the beans. After the coffee cherries are dried, the outer husk is separated from the beans by a peeling process, and the resulting husk is called a cascara husk [21,22].

The higher content of polyphenols in the Cas1 sample compared to Cas2 and Cas3 may be partly due to the fact that Cas1 was produced using the wet method, unlike the other cascara samples. This observation is consistent with published findings. Cano-Muñoz et al. investigated cascaras produced by three different methods and also found that the total phenolic content of cascara obtained through wet processing was higher than in natural processing [23].

In addition to the difference in the production process, factors such as climate, soil composition, and altitude significantly influence the phenolic compound content in the coffee cherry and cascara. These environmental factors determine the plant’s metabolic stress level, which in turn regulates the synthesis of secondary metabolites, including polyphenols (such as phenolic acids and flavonoids) [24,25].

All cascara varieties used in the study—Castillo (Cas1), Bourbon (Cas2), mixture of Caturra, and Catuai (Cas3)—are Coffea arabica cultivars (or their hybrids) widely used in the coffee industry. These varieties differ genetically, in growth characteristics, in resistance to diseases, and thus, in the content of important compounds such as polyphenols and alkaloids.

Cas1 originated from Colombia and was cultivated at an altitude of nearly 2000 m, while Cas2 and Cas3 were grown at an altitude of 1400–1500 m. Coffee cherries grown at higher altitudes generally contain a greater amount of polyphenols because the lower temperature slows down the ripening of the cherries and extends the biosynthetic period for phenolic compounds. Furthermore, higher UV radiation causes stress in the plants, triggering enhanced synthesis of antioxidant molecules (polyphenols) for self-protection. This also explains the higher content of phenolic compounds in Cas1.

Furthermore, the elevated polyphenol content in this product may also be due to the fact that Castillo is a hybrid of Coffea arabica and Coffea canephora (Robusta). Studies by Perdani et al. have also shown that Robusta coffee green beans contain more phenolic compounds than Arabica coffee green beans [26].

### 2.3. Phytochemical Analysis

Borate-based background electrolytes (BGE) are commonly used for analysis of phenolic compounds in botanical material [27]. Under basic experimental conditions in which the polyphenols become negatively charged, the separation occurs either based on charge-to-mass ratios of the deprotonated polyphenols or through borate–phenol association [28].

**Figure 1:**
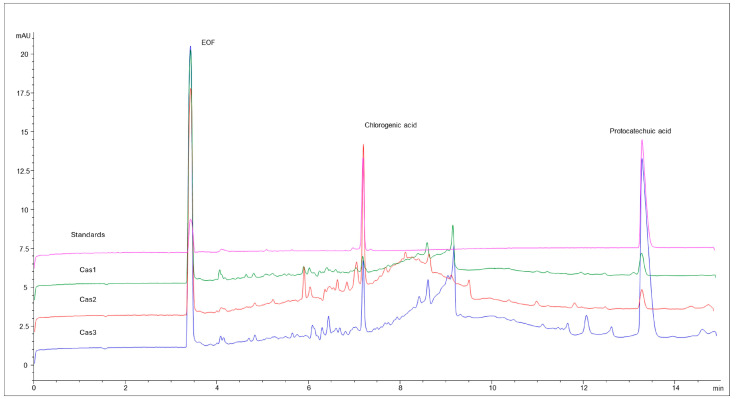
The electrophoretic fingerprint of cascara extracts. Separation conditions: BGE—25 mM sodium tetraborate, applied voltage 25 kV, detection at 254 nm. EOF—electroosmotic flow, representing uncharged extract components under the given analysis conditions.

Figure 1 presents the electrophoretic fingerprints of all three cascara extracts. Electrophoretic fingerprints are employed to characterize plant extracts by revealing the complete pattern and distribution of chemical compounds contained within the extract. This unique pattern can be used for the identification, quality control, and standardization of plant-derived extract [10].

It reflects the plant’s genetic, morphological, and chemical characteristics, which have been influenced by the growing environment (climate, soil, altitude) and processing methods [29].

As shown in Figure 1, the electropherograms of all cascara extracts are largely similar. Both Cas1 and Cas3 display a zone between 8 and 10 min, where the peaks are not baseline separated, whereas Cas2 has a broad hump. It can be speculated that tannins with a wide range of molecular weights migrate in this area. This assumption is further supported by the UV spectrum with an absorption maximum of 280 nm, which is characteristic of condensed tannins.

The two clearly resolved main peaks belong to chlorogenic and protocatechuic acids.

The electropherograms for caffeine and trigonelline in cascara samples are highly selective and do not provide broader compositional information; therefore, they are not presented here.

**Figure 2:**
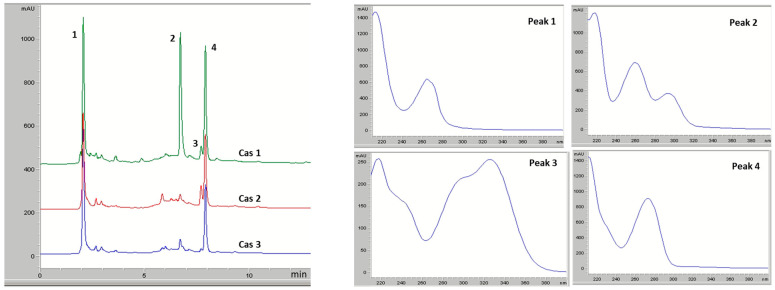
HPLC chromatograms and UV spectra of identified compounds in cascara extracts. Identification: 1—trigonelline; 2—protocatechuic acid; 3—chlorogenic acid; 4—caffeine.

Similarly to the electropherograms, the chromatograms of the cascara extracts also show a marked similarity, with caffeine and trigonelline being identified as the dominant components in all extracts (Figure 2). Their high proportion is characteristic of members of the Coffea genus and confirms that cascara retains these important coffee-related alkaloids, albeit at lower concentrations.

In addition to the standard addition method, UV-spectral comparison and mass spectrometric analysis were used to identify the peaks more characteristic of cascara (Table 3).

### 2.4. Quantification of Bioactive Compounds in Cascara Extracts

The content of the four most common bioactive substances, including two phenolic acids and two alkaloids, was determined in the cascara samples. The analytical parameters required for the quantitative analysis of caffeine, trigonelline, chlorogenic acid, and protocatechuic acid are presented in Table 4. All validation parameters were within generally accepted limits for complex plant matrices, confirming the suitability of the CE method for the analysis of cascara extracts.

Among the alkaloids, trigonelline was the most abundant in the cascara samples, ranging from 0.286 to 0.434 mg/mL (Table 5). Caffeine levels were somewhat lower, varying from 0.208 to 0.372 mg/mL. These findings are consistent with reports indicating that Coffea canephora (Robusta) contains more caffeine and trigonelline than Coffea arabica [30,31].

Bourbon, Caturra and Catuai are typical *C. arabica* varieties characterized by medium levels of caffeine and trigonelline. Castillo may produce slightly higher content of these alkaloids due to hybridization, although its levels remain lower than those typically observed in *C. canephora*.

Although the literature widely reports that chlorogenic acid is the main phenolic compound in cascara [17], its content was found to be low in the cascara samples studied. Cas1 stood out with a surprisingly high protocatechuic acid content (0.178 mg/mL), while its content was approximately 20-fold lower in both Cas2 and Cas3.

### 2.5. Antibacterial Properties

To distinguish the antibacterial activity of complex cascara extracts from that of their individual major constituents, antibacterial effects were evaluated in parallel for native aqueous extracts, pure analytical standards, and an artificial mixture formulated at concentrations equivalent to those measured in the most active extract.

#### 2.5.1. Antibacterial Activity of Cascara Extracts

The antibacterial properties of cascara extracts were evaluated using six reference bacterial strains. These strains were selected, as they are represent a major causative agent of hospital-acquired infections worldwide and frequently exhibit resistance to multiple antibiotics [1]. *S. aureus* and *E. faecium* represent Gram-positive bacteria, whereas the remaining strains are Gram-negative representatives [32].

The antibacterial activity of the cascara extracts varied among the tested bacterial strains. The resulting MBC values of cascara extracts expressed per soluble solids are summarized in Table 6.

Overall, the extracts exhibited the strongest activity against Gram-positive bacteria. For *S. aureus*, the MBC of Cas1 and Cas3 were similar, 0.75 and 1.65 mg/mL, respectively, whereas Cas2 showed a substantially higher MBC of 7.09 mg/mL. A similar trend was observed for the second Gram-positive bacterium, *E. faecium*, with MBCs of 2.99 and 3.30 mg/mL for Cas1 and Cas3, and a higher value of 7.09 mg/mL for Cas2.

Among the Gram-negative bacteria, *K. pneumoniae* and *E. cloacae* were the least sensitive to the cascara extracts. Interestingly, relatively low MBC values were observed for *P. aeruginosa*, with 5.97 and 3.3 mg/mL for Cas1 and Cas3, respectively, while Cas2 showed a consistently high MBC of 28.37 mg/mL.

The most notable results were obtained for *A. baumannii*, a Gram-negative pathogen. Cas1 exhibited a remarkably low MBC of 0.75 mg/mL, similar to that observed for *S. aureus*. Cas2 and Cas3 also demonstrated substantial antibacterial activity against *A. baumannii*, with MBCs of 3.3 and 7.09 mg/mL, respectively.

Overall, these findings indicate that cascara extracts exert a pronounced effect on Gram-positive bacteria, while certain extracts, particularly Cas1, also display notable activity against certain Gram-negative strains, most prominently *A. baumannii* and *P. aeruginosa*.

Similar findings were reported by Tasew et al. [33], where the antibacterial activities of different coffee samples from the same geographical region were shown to be variable. In that study, although disk diffusion assays indicated that *S. aureus* was the most susceptible to the antibacterial effects of coffee, the MBC values for *S. aureus* and *P. aeruginosa* were identical, both measured at 62.50 mg/mL. These observations are in line with our results, which likewise demonstrate pronounced activity of cascara extracts against Gram-positive bacteria, while certain Gram-negative strains, such as *P. aeruginosa*, exhibited moderate susceptibility depending on the specific extract. Almeida et al. [13] demonstrated that natural coffee compounds and coffee extract exhibit activity against *Streptococcus mutans*. When trigonelline, caffeine, caffeic acid, and protocatechuic acid were incorporated into the coffee extract, they caused a marked increase in the antimicrobial effect compared to the original extract.

#### 2.5.2. Antibacterial Activity of Standards

Caffeine is one of the most widely consumed alkaloids globally and possesses well-characterized pharmacological effects along with an established safety profile. It is generally regarded as safe for daily intakes up to 400 mg. However, when evaluated individually, caffeine demonstrates only modest antibacterial activity. In studies conducted on clinical isolates of *S. aureus* with varying antibiotic resistance patterns, measurable antibacterial effects were only observed at relatively high concentrations, reaching approximately 16 mg/mL for most strains [34]. However, recent MBC analysis has indicated that caffeine standard has a stronger inhibitory value against tested bacteria. Those results are depicted by the lower MBC value of the caffeine standard with a range of 0.125 and 0.250 mg/mL against *S. aureus* and *P. aeruginosa*, respectively [35]. In our experiment, we likewise determined the MBC of caffeine against *S. aureus* to be 0.125 mg/mL. However, no antibacterial activity was observed against any of the other tested bacterial strains (Table 7). Differences between reported values may stem from variations in the bacterial strains used (clinical isolates versus reference strains), methodological discrepancies (broth-based versus agar-based assays), as well as differing criteria applied for defining MBC.

Previous studies have reported that catechin generally possesses very limited antibacterial potential. Its activity against *P. aeruginosa*, *Escherichia coli*, and *S. aureus* was reported to correspond to MIC values as high as 1024 mg/mL for all tested strains [36]. In contrast, markedly stronger antibacterial effects of catechin have been observed against clinical isolates of *S. aureus*, with MIC values ranging from 0.078 to 0.156 mg/mL [37]. In our study, the MBC of catechin for *S. aureus* was determined to be 0.5 mg/mL and no antibacterial activity of catechin was detected against any of the other tested bacterial strains.

Protocatechuic acid (PCA) is known in the literature to produce antibacterial effects due to its ability to inhibit bacterial growth. Its antibacterial efficacy against both Gram-positive and Gram-negative bacteria has been demonstrated in several studies. For instance, Kuete et al. reported an MBC value of 0.31 mg/mL for the Gram-negative bacteria *P. aeruginosa*, while no bactericidal effect was observed against *K. pneumoniae*. Interestingly, the MBC for the Gram-positive strain *S. aureus* was also reported to be 0.31 mg/mL in that study [38]. Moreover, another in vitro investigation proves that PCA addition can significantly enhance the antibacterial activity of commonly used drugs, such as levofloxacin, nitrofurantoin, and cotrimoxazole, against both Gram-positive and Gram-negative bacterial strains increasing their efficacy by up to 50% [39].

In our investigation, PCA exhibited stronger bactericidal activity against Gram-positive bacteria, with MBC values of 0.125 mg/mL and 0.025 mg/mL for *S. aureus* and *E. faecium*, respectively (Table 6). Among the Gram-negative bacteria tested, PCA showed an MBC of 0.2 mg/mL for all species except *A. baumannii*, which demonstrated resistance to PCA under the conditions of our study.

Chlorogenic acid (CGA) is a phenolic compound broadly distributed in plants and widely investigated for its antimicrobial potential. CGA was tested for its antibacterial activity against clinical isolates of *Stenotrophomonas maltophilia* and demonstrated clear bactericidal effects, with MBC values ranging consistently from 0.016 to 0.032 mg/mL [40]. In a subsequent study, CGA and its isomers isolated from *Stevia rebaudiana* were evaluated for antibacterial activity. All six isomers exhibited inhibitory effects against the tested strains, showing greater efficacy against Gram-negative than Gram-positive bacteria. Among the tested strains, *E. coli* was the most sensitive to the extracts. Notably, 3-CGA and iso-chlorogenic acid C (ICAC) demonstrated the highest antibacterial activity, with MICs of 2 mg/mL and 4 mg/mL against *E. coli* ATCC 25922 and MDR *E. coli*, respectively [41].

In our study, Gram-positive bacteria, *S. aureus* and *E. faecium*, were more susceptible to CGA, with MBC values of 0.125 mg/mL and 0.25 mg/mL, respectively (Table 6). Most of the tested Gram-negative bacteria were insensitive to CGA, with the exception of *A. baumannii*, which exhibited an MBC of 0.5 mg/mL. These findings are consistent with previously published data, where MIC values against Gram-negative species were substantially higher than the concentrations used in our study.

Tannic acid (TA) exhibited notable bactericidal activity against both Gram-positive and Gram-negative bacteria, although the efficacy varied considerably depending on the species and strain. In vitro studies demonstrated an MBC of 0.7 mg/mL against *Salmonella typhimurium* [42], while tests on skin-associated bacterial isolates showed a broader range of MBC values, from 0.25 mg/mL to >16 mg/mL, reflecting strain-specific susceptibility [43]. Moreover, TA has been reported to inhibit biofilm formation and disrupt existing biofilms in methicillin-resistant *S. aureus* (MRSA), suggesting that its antibacterial effects may extend beyond planktonic cells to biofilm-associated populations [44].

In the present study, Gram-positive bacteria, *S. aureus* and *E. faecium*, showed higher susceptibility to TA, exhibiting MBC values of 0.016 mg/mL and 0.5 mg/mL, respectively (Table 6). In contrast, most of the tested Gram-negative species were largely resistant to TA, with the notable exception of *A. baumannii*, which demonstrated an MBC of 0.5 mg/mL.

Limited information is available regarding the antibacterial properties of trigonelline, particularly against clinically relevant and hospital-associated pathogens. Available in vitro studies indicate that trigonelline exhibits moderate antibacterial activity, with MIC values ranging from 1.28 to 5.12 mg/mL and corresponding MBC values from 2.56 to 10.24 mg/mL for oral pathogens such as *Streptococcus parasanguinis*, *Porphyromonas gingivalis*, *Fusobacterium nucleatum*, *Prevotella intermedia*, and *Prevotella nigrescens* [45]. Coffee extracts enriched with trigonelline have been shown to significantly enhance antimicrobial effects. It was reported that trigonelline, among other coffee-derived compounds, improved the inhibition of Enterobacteriaceae strains when compared to untreated extracts, although exact MBC values were not disclosed [13].

Among all the tested standards, trigonelline exhibited the strongest antibacterial activity against the bacterial stains included in our study. Notably, its MBC values against Gram-positive bacteria, *S.F aureus* and *E. faecium*, were 0.016 mg/mL and 0.063 mg/mL, respectively (Table 6). Although *K. pneumoniae* and *P. aeruginosa* were largely insensitive to trigonelline at the tested concentrations, the MBC values for the other Gram-negative bacteria, *A. baumannii* and *E. cloacae*, were 0.063 mg/mL and 0.5 mg/mL, respectively. Our results are highly consistent with those reported by Almeida et al. Among the tested compounds, *E. cloacae* were found to be the most sensitive to chlorogenic acid, protocatechuic acid, and trigonelline [46].

Our results across all tested standards indicate a more pronounced antibacterial activity against Gram-positive bacteria, where we determined MBC for every compound against *S. aureus* and for four standards against *E. faecium*. In contrast, the Gram-negative bacteria generally exhibited lower susceptibility: *K. pneumoniae* and *P. aeruginosa* were sensitive only to protocatechuic acid under our experimental conditions. Notably, both protocatechuic acid and trigonelline exhibited clear antibacterial effects against *E. cloacae*. Surprisingly, *A. baumannii*—a pathogen of major clinical concern due to its notorious multidrug resistance, biofilm formation capabilities, and prevalence in hospital-acquired infections (especially in intensive care units) [47] proved to be sensitive to tannic acid, chlorogenic acid, and trigonelline. Importantly, trigonelline displayed a very low MBC value against *A. baumannii* (0.063 mg/mL), underscoring its potential as a promising agent against this critical nosocomial pathogen.

The analysis of bioactive compounds in cascara extracts revealed that trigonelline was present at the highest concentration and correspondingly exhibited the strongest and broadest antibacterial activity, particularly against Gram-positive strains and select Gram-negative bacteria (Table 4 and Table 6). Caffeine, while the second most abundant compound, showed limited antibacterial effect, and was active only against *S. aureus*. Protocatechuic acid demonstrated notable bactericidal activity against Gram-positive bacteria and moderate effects against most Gram-negative species, whereas chlorogenic acid was primarily effective against Gram-positive bacteria, with limited activity against *A. baumannii*. These results suggest that the antibacterial activity of cascara extracts may be largely attributed to trigonelline, while other compounds appear to exert more targeted, species-dependent effects. However, the variation in activity highlights the synergistic potential of multiple bioactive constituents in the extracts.

To better understand the antibacterial properties of the aqueous cascara extracts, the MBC values against the tested bacteria were recalculated based on the concentrations of individual bioactive compounds present in the extracts. The resulting data are presented in Table 8. These recalculated values were subsequently compared with the MBCs of the pure standards (Table 7) to assess the contribution of each individual compound to the overall antibacterial activity of the extracts.

Interestingly, despite containing the same active compounds at substantially lower individual concentrations, the aqueous extracts (Cas1–Cas3) exhibited unexpectedly enhanced antibacterial activity. For instance, the total MBC of the Cas1 extract against *S. aureus* and *A. baumannii* was 0.03 mg/mL, lower than any of the individual standards, despite the lower concentration of each compound in the mixture. Cas1 also displayed a total MBC of 0.19 mg/mL against *E. faecium*, 1.035 mg/mL against *K. pneumoniae*, 0.26 mg/mL against *P. aeruginosa*, and 0.52 mg/mL against *E. cloacae*, indicating that the extract retained broad-spectrum activity. Similarly, Cas2 exhibited total MBC values of 0.15 mg/mL for *S. aureus* and *E. faecium*, 0.58 mg/mL for both *K. pneumoniae* and *P. aeruginosa*, 0.145 mg/mL for *A. baumannii*, and 0.581 mg/mL for *E. cloacae*, confirming enhanced antibacterial effects compared to individual compounds. Cas3 demonstrated particularly strong activity, with total MBCs of 0.039 mg/mL for *S. aureus*, 0.078 mg/mL for *E. faecium*, 0.622 mg/mL for *K. pneumoniae*, 0.029 mg/mL for *P. aeruginosa*, 0.029 mg/mL for *A. baumannii*, and 0.622 mg/mL for *E. cloacae*. Notably, even for Gram-negative bacteria, which were generally less sensitive to individual standards, the combined extracts substantially lowered MBC values, demonstrating synergistic or additive effects of trigonelline, protocatechuic acid, chlorogenic acid, caffeine, and other minor constituents. Recalculation of MBC values based on individual compound concentrations demonstrated that the bactericidal activity of native cascara extracts cannot be explained by the measured levels of their major bioactive components alone. This observation indicates that additional constituents and interactions within the extracts play a substantial role in shaping antibacterial effects. Overall, these findings indicate that complex extracts, despite containing lower concentrations of individual active molecules, can outperform pure standards across multiple bacterial species, highlighting their potential for natural antimicrobial applications and suggesting a promising strategy for targeting both Gram-positive and Gram-negative pathogens, including clinically relevant multidrug-resistant strains such as *A. baumannii*.

#### 2.5.3. Antibacterial Activity of Artificial Mixture

**Figure 3:**
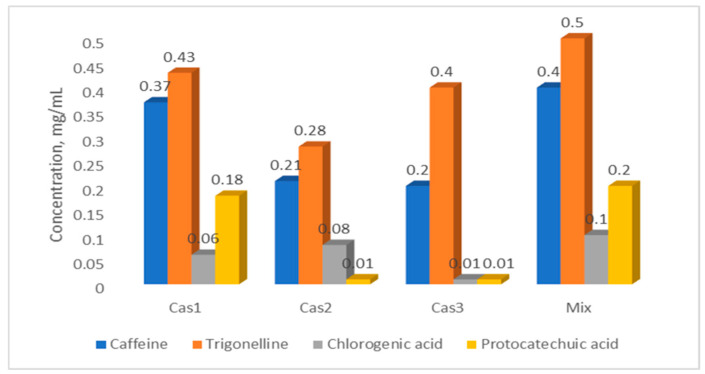
Concentrations of major bioactive compounds in cascara extracts and artificial mixture (mg/mL).

Based on the data obtained, we prepared an artificial mixture of these standards formulated to match the concentrations of their respective components in Cas1 extract. The mixture contained caffeine, trigonelline, chlorogenic acid, and protocatechuic acid at final concentrations of 0.4 mg/mL, 0.5 mg/mL, 0.1 mg/mL, and 0.2 mg/mL, respectively (Figure 3). This artificial mixture was subsequently tested for its antibacterial properties against the same panel of Gram-positive and Gram-negative bacteria used in the study.

When comparing the antibacterial efficacy of our artificial mixture to that of the native aqueous extracts (Cas1–Cas3), several patterns emerge (Table 9). For Gram-positive bacteria, both Cas1 and the artificial mixture demonstrated strong activity, with total MBC values for *S. aureus* of 0.03 mg/mL (Cas1) and 0.04 mg/mL (mix), and for *E. faecium* of 0.19 mg/mL (Cas1) and 0.15 mg/mL (mix). Against Gram-negative enteric bacteria, such as *K. pneumoniae* and *E. cloacae*, the differences were smaller: Cas1 showed total MBCs of 1.035 mg/mL and 0.52 mg/mL, while the artificial mixture exhibited slightly higher MBCs of 1.2 mg/mL and 0.6 mg/mL, respectively, and retained broad-spectrum activity despite lower complexity in the mixture.

The most notable differences were observed for *P. aeruginosa* and especially *A. baumannii*. Cas1 exhibited a substantially lower MBC against *P. aeruginosa* (0.26 mg/mL) compared to the artificial mixture (1.2 mg/mL), highlighting a strong enhancement of activity in the natural extract. For *A. baumannii*, Cas1 showed an extremely low total MBC of 0.03 mg/mL, whereas the artificial mixture, despite containing approximately three times higher concentrations of the major active compounds, displayed a higher MBC of 0.15 mg/mL. *A. baumannii* is a highly problematic nosocomial pathogen due to its multidrug resistance, robust biofilm formation, and high virulence, making effective inhibition particularly important [48]. The enhanced activity of Cas1 suggests that additional minor phytochemicals present in the natural extract may act synergistically with caffeine, trigonelline, chlorogenic acid, and protocatechuic acid, amplifying antibacterial efficacy.

In addition, other non-bactericidal compounds in cascaras such as polysaccharides, amino acids, pectic substances, or small peptides may also contribute to the inhibitory properties. Indeed, coffee cascara has been reported to contain significant amounts of pectic polysaccharides (mainly composed of homogalacturonan, with rhamnose and arabinose as minor sugars), which could influence microbial growth or modulate cell–surface interactions [12]. Moreover, cascara contains proteins and amino acid residues, as well as non-phenolic bioactive substances, which may synergize with phenolic compounds, thereby enhancing the overall antibacterial effect [49].

The observed activity may be explained by multiple mechanisms. Chlorogenic acid has been shown to disrupt bacterial membranes, increase permeability, and inhibit biofilm formation, including interference with quorum sensing in *P. aeruginosa* [50,51]. Trigonelline and protocatechuic acid, although less extensively studied, also demonstrate antibacterial activity against Gram-positive and certain Gram-negative bacteria, likely through similar membrane-targeting or enzyme-inhibiting effects [46]. Furthermore, complex polyphenolic mixtures are known to exhibit additive or synergistic effects, enhancing antibacterial activity beyond that of isolated compounds by simultaneously targeting multiple bacterial pathways, including biofilm integrity and virulence factor expression [52].

These results highlight the potential of natural cascara extracts as multi-component antimicrobial agents and suggest that the full antibacterial effect against clinically relevant multidrug-resistant pathogens, such as *A. baumannii* and *P. aeruginosa*, likely relies on synergistic interactions among both major and minor constituents. Future studies should focus on identifying these minor bioactive compounds and dissecting their combined mechanisms of action to optimize extract-based antimicrobial strategies.

## 3. Materials and Methods

### 3.1. Botanical Material and Extraction

Three commercial cascara samples used in the study were purchased from an online store. Details are given in Table 10.

Before extraction, the cascara samples were grounded using a coffee bean grinder Bomann KSW 445 CB (Bomann GmbH, Wuppertal, Germany; China). An ultrasonic-assisted method was used for extracting phytochemicals in cascara samples. Briefly, the sample powder was mixed with hot ultrapure water, with the solid-to-liquid ratio 1:20 (*w*/*v*) and sonicated at 70 °C, and 640 W (350 kHz) in Sonorex™ Digital 10P bath (Bandelin Electronic GmbH & Co. KG, Berlin, Germany). After sonication, the crude extract was centrifuged at 8000× *g* for 15 min. The supernatant was filtered through a 0.45 µm membrane and stored in a refrigerator at 4 °C for further analysis. All the experiments were performed in triplicate.

The soluble solid content of the extracts was measured by drying the sample to a constant weight in a vacuum concentrator (Eppendorf AG, Hamburg, Germany), using the gravimetric method.

### 3.2. Chemicals

Ultrapure water produced within the laboratory with a Milli-Q water purification system (Merck KGaA, Darmstadt, Germany) was used to prepare all aqueous solutions.

Total polyphenolic content of the extracts was evaluated using the 2 M Folin–Ciocalteu reagent (Sigma-Aldrich, Buchs, Switzerland) and sodium carbonate (Sigma-Aldrich, Darmstadt, Germany). Standard solutions used were prepared from gallic acid monohydrate (Sigma-Aldrich, China) in ethanol (Sigma-Aldrich, Darmstadt, Germany). The total flavonoid content was determined using aluminum chloride (Fluka, Buchs, Switzerland). Standard solutions were prepared from quercetin (Lachema/Chemapol, Brno, Czech Republic) dissolved in methanol (Honeywell, Charlotte, NC, USA).

The background electrolyte constituents, including acetic acid, disodium tetraborate decahydrate and sodium dodecyl sulfate (SDS) were purchased from Sigma-Aldrich (Germany). Commercial standards for capillary electrophoresis (CE), including caffein, trigonelline hydrochloride, chlorogenic and protocatechuic acids were purchased from Sigma-Aldrich (Darmstadt, Germany).

### 3.3. Determination of Total Polyphenol and Flavonoid Content

Two groups of bioactive compounds in the cascaras were quantified by colorimetric tests: total polyphenols by the Folin–Ciocalteu method and total flavonoids by the AlCl_3_ [53]. Both analyses were conducted on the Varian Cary 50 Bio UV-Vis spectrophotometer (Agilent Technologies, Santa Clara, CA, USA). Total polyphenolic content of the extracts was determined using calibration solutions of 10, 25, 50, 75, and 100 mg/L of gallic acid in ethanol, prepared from a 5 g/L stock solution. The calibration solutions for the quantification of flavonoid contents were prepared in concentrations of 2, 5, 10, 20, and 40 mg/L of quercetin in methanol, prepared from a 2 g/L stock solution. All samples were measured in triplicate and the results given in mg of either mean gallic acid and quercetin equivalents per L extracts (mg GAE/QE/L) ± standard deviation.

### 3.4. Capillary Electrophoretic Analysis

All analyses were performed using an Agilent Technologies 7100 CE instrument coupled with a diode-array detector (DAD). Fused silica capillaries (Polymicro Technologies, Phoenix, AZ, USA) were used throughout the study. For the analysis of caffeine optimal CE parameters, we determined the following: capillary Ltot/Lef 64/55.5 cm, I.D. 75 µm, applied voltage 20 kV, sample injection time 7 s, and sample injection pressure 35 mbar. For the analysis of phenolic acids and trigonelline, the parameters were Ltot/Lef 60/51.5 cm, I.D. 50 µm, applied voltage 25 kV, sample injection time 10 s, and sample injection at 50 mbar.

The background electrolyte (BGE) used for caffeine analysis consisted of 10 mM sodium carbonate and 50 mM SDS. The BGE for trigonelline was 1 M acetic acid, and for phenolic acids 25 mM disodium tetraborate.

Calibration solutions for caffeine and trigonelline were in the range of 0.005–0.1 mg/mL; for chlorogenic acid and protocatechuic acid 0.01–0.15 mg/mL. The cascara extracts under study were diluted as needed. The UV-absorbance detection wavelength was set to 273 nm for caffeine, 264 nm for trigonelline, and 254 nm for phenolic compounds.

### 3.5. HPLC-DAD-MS/MS Analyses

Before the HPLC-DAD-MS/MS analyses, the cascara extracts were centrifuged at 8000 rpm and diluted in ultrapure water two-fold. The injected volume was 5 µL. HPLC-DAD-MS/MS analyses were conducted using an Agilent 1260 Infinity II instrument (Agilent Technologies, Santa Clara, CA, USA) with an Agilent Poroshell 120 EC-C18 column—particle size: 2.7 µm; measurements 4.6 mm × 100 mm (Agilent Technologies, Santa Clara, CA, USA)—thermostated at 28 °C. The mobile phase consisted of ultrapure water (A) and acetonitrile (B), both acidified with 0.1% (*v*/*v*) formic acid. The elution procedure was a linear gradient increasing from 5% to 15% B (0–2 min), then from 15% to 23% B (2–10 min), isocratic 23% B (10–12 min), then linear gradient from 23% to 32% (12–16 min). The flow rate was kept at 0.5 mL/min. The column was coupled with an Infinity 1260 DAD (Agilent Technologies, Santa Clara, CA, USA), the chromatograms were recorded at the UV-absorbance wavelength of 254 nm (slit 4 nm), and DAD spectra were recorded in the range of 200 to 400 nm. Following the DAD analysis, the sample was analyzed using the LC/MSD Trap XCT mass spectrometer (Agilent Technologies, Santa Clara, CA, USA) equipped with an electrospray ionization source. The mass spectra were recorded in both the positive and negative-ion modes in the *m*/*z* range from 100 to 1000. Nitrogen was used as the nebulizing and drying gas, and helium served as the collision gas. The MS/MS fragmentation patterns (generated using the automatic Bruker Daltonics DataAnalysis 5.0 software MS/MS settings) were used to identify the compounds.

### 3.6. Bacterial Strains

The clinical isolates *S. aureus* HUMB 19594 (methicillin-resistant, MRSA) [54], *E. faecium* HUMB 65620, *K. pneumoniae* HUMB 01336, and *P. aeruginosa* HUMB 4438 D01-10 were obtained from the Human Microbiome Project culture collection (HUMB; https://eemb.ut.ee (accessed on 16 May 2025)). *E. cloacae* DSM 109592 and *A. baumannii* DSM 25645 were obtained from the German Collection of Microorganisms and Cell Cultures (DSMZ).

### 3.7. Sample Preparation for Antibacterial Assay

To investigate the antibacterial properties of cascara, three types of samples were prepared: (1) aqueous cascara extracts—Cas1, Cas2 and Cas3; (2) analytical standards of the individual compounds identified in the cascara extracts, including trigonelline, caffeine, protocatechuic acid, catechin, chlorogenic acid and tannic acid at a concentration of 0.5 mg/mL and (3) an artificial mixture of these standards formulated at concentrations equivalent to their respective levels in cascara extract 1—caffeine, trigonelline, chlorogenic acid and protocatechuic acid at concentrations 0.4 mg/mL, 0.5 mg/mL, 0.1 mg/mL and 0.2 mg/mL, respectively.

### 3.8. Minimal Bactericidal Concentration (MBC)

MBC values were determined using an agar-based drop-plating method. To evaluate the antibacterial properties, all prepared probes were serially diluted at 2-, 4-, 6-, 8-, 16-, and 32-fold. The results obtained on agar media were normalized to the content of soluble solid mg/mL of extract and also recalculated based on the concentrations of specific compounds present in the extracts.

A single colony from TSA agar plate was transferred into TSB broth and incubated for 16 h at 37 °C with agitation at 150 rpm. The overnight culture was then diluted 1:50 with fresh medium and further cultivated under the same conditions until reaching the exponential growth phase (OD_600_ = 0.6). Cells were harvested by centrifugation at 5000× *g* for 5 min, and the pellet was resuspended in an equal volume of sterile water. The pellet was finally resuspended in sterile water to achieve a cell density corresponding to OD_600_ = 0.2, which corresponds to approximately 1–2 × 10^8^ CFU/mL, depending on the strain. For the MBC assay, 100 μL of the bacterial suspension was combined with 100 μL of the compound solution and incubated at 37 °C for 24 h. After the exposure period, 3 μL aliquots of the mixture were drop-plated onto TSA agar and incubated at 37 °C for 24 h. The MBC was defined as the lowest compound concentration that resulted in no visible colony formation in the 3 μL spot. All MBC determinations were performed in three biological replicates.

## 4. Conclusions

In conclusion, our study demonstrates that cascara extracts (Cas1–Cas3) exhibit strong antibacterial activity against both Gram-positive and select Gram-negative bacteria, with the greatest efficacy observed against *S. aureus*, *E. faecium*, and *A. baumannii*. The extracts contain four major bioactive compounds—trigonelline, caffeine, protocatechuic acid, and chlorogenic acid—with trigonelline present at the highest concentration and contributing most substantially to antibacterial effects. Notably, when the MBC values were recalculated based on the concentrations of individual compounds, the complex extracts consistently outperformed the pure standards, even at lower individual concentrations, highlighting synergistic or additive interactions among the constituents. An artificial mixture of the four compounds partially reproduced this enhanced activity, further supporting the concept that combined bioactive compounds can provide broader and more potent antibacterial effects than isolated molecules. These findings underscore the potential of cascara extracts as a natural source of antimicrobial agents, capable of targeting both Gram-positive and Gram-negative pathogens, including multidrug-resistant strains such as *A. baumannii*, and suggest promising applications for food safety and clinical settings.

## Figures and Tables

**Table 1:** Total soluble solid contents of cascara extracts (mg/mL).

Cas1	Cas2	Cas3
23.94 ± 0.89 mg/mL	28.37 ± 0.59 mg/mL	26.40 ± 0.21 mg/mL

**Table 2:** Total polyphenols and flavonoid concentrations of cascara extracts.

Sample	Total Polyphenols,	Total Flavonoids,
	mg GAE/L ± SDV	mg QE/L ± SDV
Cas1	802.2 ± 23.4	134.7 ± 13.2
Cas2	403.5 ± 18.6	57.3 ± 4.6
Cas3	536.8 ± 36.6	96.5 ± 11.0

**Table 3:** Identification of main compounds in cascara extracts by HPLC-DAD-MS/MS.

Peak No	RT, Min	λ_max_	MW	[M+H]^+^/ [M−H]^−^	Fragmentation	Identification
1	2.05	216, 264	137	138/-	-	Trigonelline
2	6.67	218, 269, 294	154	-/153	109	Protocatechuic acid
3	7.68	218, 326	354	-/353	191	Chlorogenic acid
4	7.89	207, 273	194	195/-	109	Caffeine

**Table 4:** Analytical parameters for quantifying bioactive compounds in cascara extracts.

Compound	Equation of Regression	LOD/LOQ, mg/mL	Repeatability/ Reproducibility *, %	Recovery *, %	R^2^	Linear Range mg/mL
Caffeine	y = 2838x − 2.099	0.001–0.003	≤4.4/≤8.9	86.2–102.8	0.9986	0.005–0.1
Trigonelline	y = 886.7x + 1.9317	0.003–0.0011	≤4.9/≤8.3	87.6–103.1	0.9999	0.005–0.1
Chlorogenic acid	y= 0.7104x + 1.6426	0.008–0.026	≤5.1/≤11.2	83.7–99.8	0.9936	0.01–0.15
Protocatechuic acid	y = 1.8598x + 1.896	0.005–0.015	≤3.8/≤8.3	89.4–102.0	0.9966	0.01–0.15

* Values were calculated based on the results obtained for Cas1, Cas2, and Cas3 samples.

**Table 5:** Concentrations of major bioactive compounds in cascara extracts (mg/mL).

Sample	Caffeine	Trigonelline	Chlorogenic Acid	Protocatechuic Acid
Cas1	0.372 ± 0.031	0.434 ± 0.048	0.057 ± 0.002	0.178 ± 0.007
Cas2	0.211 ± 0.022	0.286 ± 0.029	0.083 ± 0.006	0.008 ± 0.001
Cas3	0.208 ± 0.026	0.407 ± 0.047	0.010 ± 0.001	0.012 ± 0.001

**Table 6:** MBC values of cascara extracts for soluble solid extracts (mg/mL).

Sample	*S. aureus*	*E. faecium*	*K. pneumoniae*	*P. aeruginosa*	*A. baumannii*	*E. cloacae*
Cas1	0.75	2.99	23.89	5.97	0.75	11.95
Cas2	7.09	7.09	28.37	28.37	7.09	28.37
Cas3	1.65	3.30	26.40	3.30	3.30	26.40

**Table 7:** MBC values of standards (mg/mL).

Standard	*S. aureus*	*E. faecium*	*K. pneumoniae*	*P. aeruginosa*	*A. baumannii*	*E. cloacae*
Protocatechuic acid	0.125	0.025	0.200	0.200	-	0.200
Catechin	0.500	-	-	-	-	-
Chlorogenic acid	0.125	0.25	-	-	0.500	-
Trigonelline	0.016	0.063	-	-	0.063	0.500
Caffeine	0.125	-	-	-	-	-
Tannic acid	0.016	0.500	-	-	0.500	-

**Table 8:** MBC values of cascara extracts expressed as concentrations of major bioactive compounds (mg/mL).

	*S. aureus*	*E. faecium*	*K. pneumoniae*	*P. aeruginosa*	*A. baumannii*	*E. cloacae*
Cas1_caff	0.010	0.050	0.370	0.090	0.010	0.190
Cas1_trig	0.010	0.050	0.430	0.110	0.010	0.220
Cas1_proto	0.010	0.020	0.178	0.040	0.010	0.090
Cas1_chl	0.000	0.070	0.057	0.010	0.000	0.030
Sum	0.030	0.190	1.035	0.250	0.030	0.530
Cas2_caff	0.050	0.050	0.210	0.210	0.053	0.210
Cas2_trig	0.070	0.070	0.280	0.280	0.070	0.280
Cas2_proto	0.000	0.000	0.008	0.008	0.002	0.008
Cas2_chl	0.020	0.020	0.083	0.083	0.021	0.083
Sum	0.150	0.150	0.581	0.581	0.146	0.581
Cas3_caff	0.013	0.025	0.200	0.025	0.025	0.200
Cas3_trig	0.025	0.050	0.400	0.002	0.002	0.400
Cas3_proto	0.001	0.002	0.012	0.002	0.002	0.012
Cas3_chl	0.001	0.001	0.010	0.001	0.001	0.010
Sum	0.039	0.078	0.622	0.030	0.030	0.622

Sum—total of standard compounds.

**Table 9:** MBC values of an artificial mixture expressed as concentrations of major bioactive compounds (mg/mL).

	*S. aureus*	*E. faecium*	*K. pneumoniae*	*P. aeruginosa*	*A. baumanii*	*E. cloacae*
Mix_caff	0.013	0.050	0.400	0.400	0.050	0.200
Mix_trig	0.016	0.063	0.500	0.500	0.063	0.250
Mix_proto	0.006	0.025	0.200	0.200	0.025	0.100
Mix_chl	0.003	0.013	0.100	0.100	0.013	0.050
Mix_sum	0.040	0.150	1.200	1.200	0.150	0.600

**Table 10:** Origin, production process and variety of cascara used in the study.

	Customer	Variety	Processing	Origin
Cas1	Shokunin coffee collective, The Netherlands	Castillo	Fully washed	Columbia
Cas2	Green plantation, Czech Republic	Bourbon	Natural	Panama
Cas3	Gust, Belgium	Caturra Catuai	Natural	Costa Rica

## Data Availability

The original contributions presented in this study are included in the article. Further inquiries can be directed to the corresponding author.

## Abbreviations

The following abbreviations are used in this manuscript:

Cas	Cascara
ESKAPE	*Enterococcus faecium*, *Staphylococcus aureus*, *Klebsiella pneumoniae*, *Acinetobacter baumannii*, *Pseudomonas aeruginosa* and *Enterobacter* spp.
MBC	Minimal bactericidal concentration
MIC	Minimal inhibitory concentration
QE/L	Quercetin equivalent per liter
MDR	Multi-drug resistant
BGE	Background electrolyte
DAD	Diode-array detector
PCA	Protocatechuic acid
CGA	Chlorogenic acid
TA	Tannic acid
MRSA	Methicillin-resistant *S. aureus*
